# Stem cambial variants of Taiwan lianas

**DOI:** 10.1186/s40529-022-00358-5

**Published:** 2022-09-24

**Authors:** Sheng-Zehn Yang, Po-Hao Chen, Jian-Jhong Chen

**Affiliations:** 1 Department of Forestry, National Pingtung University of Science and Technology, No. 1, Shuefu Rd., Neipu, Pingtung, 91201 Taiwan; 2 Graduate Institute of Bioresources, National Pingtung University of Science and Technology, No. 1, Shuefu Rd., Neipu, Pingtung, 91201 Taiwan; 3 Luodong Forest District Office, Taiwan, No. 118, Zhongzheng North Rd., Luodong Town, 263 Yilan Taiwan

**Keywords:** Centrifugal xylem, Lianas, Parenchymatization, Secondary growth, Taiwan

## Abstract

**Background:**

Cambium in lianas, responsible for secondary growth, develop diverse and diagnostic traits during the climbing phase. Studies on the cross-section of Taiwanese liana cambial variants are scarce. We collected multiple stem cross-sections from 287 liana species belonging to 52 families. Each sample was examined on five occasions, and the observations were documented.

**Results:**

The results showed that approximately 22 cambial variants types were displayed in Taiwan lianas. Among these, axial vascular elements in radial segments were the most common, followed by the variants with the irregular conformation and intraxylary phloem. Based on our assessment, we provide the following identification features of a few families: Apocynaceae had intraxylary phloem; Convolvulaceae had intraxylary phloem combined with successive cambia; Lardizabalaceae, Menispermaceae, and Ranunculaceae possessed axial vascular elements in segments; Piperaceae had external primary vascular bundle cylinder combined with axial vascular elements in segments; Vitaceae had axial vascular elements in segments combined with irregular conformation. Axial vascular elements in segments and intraxylary phloem appeared in six or five combination types, showing that these two types combined with many variants are helpful for the identification of lianas. Two species, *Momordica charantia* var. *abbreviata,* and *Momordica cochinchinensis* had a cambium element in the outer cylinder of cortical bicollateral vascular bundles and formed directional layers of successive cambia.

**Conclusions:**

Our study documented regular secondary growth with a single cambium in 36 species and cambial variants present in 16 species of Taiwanese lianas. Furthermore, we provide crucial baseline data on liana cambial variations, thereby improving our understanding of their morphology and identification.

## Background

The climbing plants can be divided into lianas and herbaceous vines based on the degree of stem lignification. Lianas, a group of perennial climbing shrubs, have fibrous, thick, and truly lignified stems. On the contrary, herbaceous vines have slender and herbaceous stems (Gentry 1991; Putz and Mooney 1991). Most lianas grow without any external stem support, gradually forming unique stem variations. These variations originate in the cambium, changing the shape and structure of the stems into irregular forms, called cambial variants (Carlquist 2001). The differences in these structures can be used to distinguish liana families (Caballé 1993; Angyalossy et al. 2012).

The stem of several lianas species begins with stiff searching branches or a self-supporting shrub structure. Transverse sections of the stem from the pith to the cambium revealed that the inner secondary xylem of the self-supporting phase is characterized by the presence of shrub-type xylem, a few narrow vessels, and thick fibers. However, the xylem of the ensuing, non-self-supporting (climbing) phase is characterized by very wide vessels, low density, and intermixed soft and stiff tissues referred to as liana-type xylem.

The characteristics of lianescent vascular syndrome in lianas are related to the secondary growth, which results in special attributes, such as wide conducting cells in xylem and phloem, high abundance of parenchyma, fewer fibers, wide rays, and variations in the cambium (Angyalossy et al. 2015; Dias-Leme et al. 2021). Numerous studies have been conducted on the secondary growth of liana stems of different families (Metcalfe and Chalk 1985; Carlquist 1991, 2001, 2007, 2013; Caballé 1993; Jansen et al. 2002; Acevedo-Rodríguez 2005; Isnard and Silk 2009; Angyalossy et al. 2012, 2015; Pace et al. 2018). In Taiwan, liana cambial variants of stem cross-sections were studied in Fabaceae (Yang et al. 2016), Menispermaceae (Yang and Chen 2016), Piperaceae (Yang and Chen 2017), Lardizabalaceae and Sabiaceae (Yang et al. 2019), Convolvulaceae (Yang et al. 2020), Ranunculaceae (Yang et al. 2021), and in families with xylem in plate type (Yang and Chen 2015). Carlquist (2001) and Angyalossy et al. (2015) listed the angiosperm orders and families of climbing plants that possess the cambial variants, promoting the identification of certain families and genera.

Taiwan is located in the subtropical monsoon region; the climate is warm and humid throughout the year, approximately 22–24 °C, and annual average rainfall is about 2000–2500 mm precipitation. Due to the favorable climate, diverse species, including climbing plants, naturally occur in this region. Approximately 553 species of climbing plants belonging to 65 families are documented in Taiwan, accounting for approximately 11% of the native flora (Stevens 2001). Of these, 101 plants (23% of the climbing plants in Taiwan) are endemic to this region, indicating a high degree of endemism. Sixty one percent of the climbers (337 species) are lianas, of which 62 (18.3% of lianas) are endemic. The remaining 39% (216 species) is composed of vines, of which 39 (18.1% of vines) are endemic. In this study, we investigated the cambial variants of Taiwan lianas families. Our results contribute to the classification and ecology of climbing plants and ultimately integrate with the conservation research of global vine diversity.

## Materials and methods

We collected plant stems of various sizes from 287 lianas from 52 families in the different habitats of Taiwan (Table 1), to observe the vascular bundle development in stem cross-sections. The samples were collected at 1.3 m height to obtain comparable measurements of the diameter at breast height. The fresh materials were divided into approximately 5 cm long pieces, and a flat cross-section of each stem was cut using a cutter blade. We immediately took pictures of the stem cross sections using a Nikon D80 SLR digital camera (Lens AF Micro Nikon 60 mm 1: 2.8D, Nikon Corporation, Tokyo, Japan), and qualitative and quantitative anatomical traits were determined using Image-J (v 1.50 h) software (Ferreira and Rasband 2011). All our photographs are made from macroscopic observations. The specimens were dried in an oven (60 °C) for 4–5 days and then stored at − 20 °C for 3–4 days. Liana species were identified using a field guide (Boufford et al. 2003). All the collected specimens were deposited in the Provincial Pingtung Institute herbarium (PPI) at the National Pingtung University of Science and Technology, Pingtung, Taiwan, for subsequent identification.

**Table 1:** Cambial variants of each species in Taiwan lianas

Families	Scientic name	Cambial variants
Acanthaceae	*Thunbergia alata* Bojer ex Sims	FD
	*Thunbergia grandiflora* Roxb	TE
	*Thunbergia laurifolia *Lindl	TE
Actinidiaceae	*Actinidia arguta* (Sieb. & Zucc.) Planch. ex Miquel	RC
	*Actinidia callosa* var. *discolor* C. F. Liang Feng	RC
	*Actinidia latifolia* (Gardner & Champ.) Merr	RC
	*Actinidia rufa* (Sieb. & Zucc.) Planch. ex Miquel	RC
	^a^*Actinidia setosa* (H. L. Li) C. F. Liang & A. R. Ferguson	RC
Amaranthaceae	*Deeringia amaranthoides* (Lam.) Merr	SC
Anacardiaceae	*Rhus ambigua* Lav. ex Dippel	RC
Annonaceae	*Artabotrys hexapetalus* (Linnaeus f.) Bhandari	RC
	*Fissistigma glaucescens* (Hance) Merr	RC
	*Fissistigma oldhamii* (Hemsl.) Merr	RC
Apocynaceae	*Alyxia sibuyanensis* Elmer	TR
	^a^*Alyxia taiwanensis* S. Y. Lu & Yuen P. Yang	TR
	*Anodendron affine* (Hook. & Arn.) Druce	TR
	^a^ *Anodendron benthamiana* Hemsl	TR
	*Cryptolepis sinensis* (Lour.) Merr	TR
	*Dregea volubilis* (L. f.) Benth	TR
	*Gymnema sylvestre* (Retz.) Schultes	TR, FC
	^a^*Heterostemma brownii* Hayata	TR, FC
	*Marsdenia formosana* Masam	TR
	*Marsdenia tinctoria* R. Br	TR
	^a^ *Melodinus angustifolius* Hayata	TR
	*Parsonsia alboflavescens *(Dennst.) Mabb	TR
	^a^ *Trachelospermum formosanum* Y. C. Liu & C. H. Ou	TR
	*Trachelospermum gracilipes* Hook. f	TR, ES
	*Trachelospermum jasminoides* (Lindl.) Lemaire	TR, FC
	^a^*Trachelospermum lanyuense* C. E. Chang	TR
	*Urceola micrantha* (Wallich *ex* G. Don) D. J. Middleton	TR
	*Urceola rosea* (Hook. & Arn.) D. J. Middleton	TR
Araliaceae	*Eleutherococcus trifoliatus* (L.) S. Y. Hu var. *setusus* (H. L. Li) H. Ohashi	IC
	*Eleutherococcus trifoliatus* (L.) S. Y. Hu var. *trifoliatus*	RC
	^a^*Hedera rhombea* (Miq.) Bean var. *formosana* (Nakai) H. L. Li	RC
Areacaceae	^a^*Calamus formosanus* Becc	RC
	^a^*Calamus siphonospathus* Martius	RC
Aristolochiaceae	^a^*Aristolochia cucurbitifolia* Hayata	VS
	*Aristolochia elegans* Mast	VS
	*Aristolochia shimadae* Hayata	VS
	*Aristolochia zollingeriana* Miq	VS
Asteraceae	*Blumea riparia* (Blume) DC. var. *megacephala* Randeria	VS
	*Microglossa pyrifolia* (Lam.) Kuntze	IC
	*Mikania micrantha* Kunth	FD
	*Senecio scandens* Buch.-Ham. ex D. Don var. *scandens*	RC
	*Vernonia gratiosa* Hance	VS
Basellaceae	*Anredera cordifolia* (Tenore) van Steenis	VS, SC
Bignoniaceae	*Anemopaegma chamberlaynii* (Sims) Bureau & K.Schum	FD
	*Pyrostegia venusta* (Ker-Gawl.) Miers	FD
Cannabaceae	*Humulus scandens* (Lour.) Merr	IC
Capparaceae	^a^*Capparis formosana* Hemsl	RC
	*Capparis lanceolaris* DC	RC
Caprifoliaceae	*Lonicera acuminata* Wall	RC
	*Lonicera hypoglauca* Miq	RC
	*Lonicera japonica* Thunb	RC
Cecropiaceae	*Poikilospermum acuminata* (Trécul) Merr	RC
Celastraceae	*Celastrus hindsii* Benth	FC
	*Celastrus kusanoi* Hayata	VS
	*Celastrus paniculatus* Willd	RC
	*Celastrus punctatus* Thunb	FC
	^a^*Euonymus spraguei* Hayata	IC
	*Tripterygium wilfordii* Hook. f	RC
Combretaceae	*Quisqualis indica* L	TR, FC
Connaraceae	*Rourea minor* (Gaertn.) Leenhouts	SC
Convolvulaceae	^a^*Argyreia akoensis* S. Z. Yang, P. H. Chen & G. W. Staples	TR, SC
	^a^*Argyreia formosana* Ishigami ex T. Yamaz	TR, SC
	*Argyreia nervosa* Boj	TR, SC
	*Camonea vitifolia* (Burm.f.) A. R. Simões & Staples	TR, SC
	*Distimake quinquefolius* (L.) A.R.Simões & Staples	TR
	*Distimake tuberosus* (L.) A.R.Simões & Staples	TR, SC
	*Erycibe henryi* Prain	TR, SC
	*Ipomoea alba* L	TR, SC
	*Ipomoea batatas* (L.) Lam	TR, SC
	*Ipomoea cairica* (L.) Sweet	TR, SC
	*Ipomoea carnea* Jacq. subsp*. fistulosa* (Mart. ex Choisy) D. F. Austin	TR
	*Ipomoea hederifolia* L	TR, SC
	*Ipomoea indica* (Burm. f.) Merr	TR, SC
	*Ipomoea littoralis* Blume	TR, SC
	*Ipomoea nil* (L.) Roth	TR, SC
	*Ipomoea obscura* (L.) Ker Gawl	TR, SC
	*Ipomoea pes-caprae* (L.) R. Br. subsp. *brasiliensis* (L.) Oostst	TR, SC
	*Ipomoea quamoclit* L	TR
	*Ipomoea triloba* L	TR, SC
	*Ipomoea violacea* L	TR
	*Lepistemon binectariferum* (Wall.) Kuntze var. *trichocarpum* (Gagnep.) Ooststr	TR, SC
	*Merremia gemella* (Burm.f.) Hallier f	TR, SC
	*Operculina turpethum* (L.) S. Manso	TR, SC
	*Stictocardia tilifolia* (Desr.) Hallier f	TR, SC
Cucurbitaceae	*Coccinia grandis* (L.) Voigt	IC, VS
	*Gynostemma pentaphllum* (Thunb.) Makino	IC, VS
	*Melothria pendula* L	IC, VS
	*Momordica charantia* L	IC, VS, SC
	*Momordica charantia* L. var. *abbreviata* Ser	IC, VS, SC
	*Momordica cochinchinensis* (Lour.) Spreng	FD, VS, SC
	*Neoalsomitra integrifolia* (Cogn.) Hutch	FD, VS
Elaeagnaceae	*Elaeagnus formosana* Nakai	RC
	*Elaeagnus glabra* Thunb	RC
	^a^*Elaeagnus grandifolia* Hayata	RC
	^a^*Elaeagnus thunbergii* Serv	RC
	*Elaeagnus triflora* Roxb	RC
Euphorbiaceae	*Mallotus repandus* (Willd.) Müll. Arg	IC
Fabaceae	*Abrus precatorius* L	IC
	*Bauhinia championii* (Benth.) Benth	DX, IC
	*Caesalpinia bonduc* (L.) Roxb	RC
	*Caesalpinia crista* L	RC
	*Caesalpinia decapetala* (Roth) Alston	RC
	*Caesalpinia minax* Hance	RC
	*Callerya nitida* (Benth.) R. Geesink	RC
	*Calopogonium mucunoides* Desv	IC
	*Canavalia cathartica* Thouars	RC
	*Canavalia lineata* (Thunb.) DC	RC
	*Canavalia rosea* (Sw.) DC	RC
	*Centrosema pubescens* Benth	FC
	*Clitoria ternatea* L	RC
	*Dalbergia benthamii* Prain	RC
	^a^*Derris laxiflora* Benth	RC
	*Derris trifoliata* Lour	RC
	^a^*Dolichos rilobus* L. var. *kosyunensis* (Hosok.) H. Ohashi & Tateishi	FC
	*Dunbaria merrillii* Elmer	RC
	^a^*Dumasia truncata* Siebold & Zucc	RC
	*Entada phaseoloides* (L.) Merr. subsp. *phaseoloides*	SC
	*Entada phaseoloides* (L.) Merr. subsp. *tonkinensis* (Gagnep.) H. Ohashi	SC
	*Entada rheedei* Spreng	SC
	*Lablab purpureus* (L.)Sweet	ES, IC
	*Macroptilium atropurpureum* (DC.) Urb	RC
	*Macroptilium lathyroides* (L.) Urb	RC
	*Millettia pachycarpa* Benth	RC
	*Mimosa diplotricha* C. Wright ex Sauvalle	FC
	*Mucuna gigantea* (Willd.) DC.subsp. *tashiroi* (Hayata) H. Ohashi &Tateishi	RC
	*Mucuna macrocarpa* Wall	SC
	*Mucuna membranacea* Hayata	RC
	*Mucuna pruriens* (L.) DC. var. *utilis* (Wall. ex Wight) Burck	IC
	*Neonotonia wightii* (Wight & Arn.) Lackey	RC
	*Paraderris canarensis* (Dalzell) Adema	RC
	*Paraderris elliptica* (Wallich) Adema	RC
	*Psophocarpus tetragonolobus* (L.) DC	RC
	*Pueraria lobata* (Willd.) Ohwi subsp. *thomsonii* (Benth.) Ohashi & Tateishi	SC
	*Pueraria montana* (Lour.) Merr	SC
	*Senegalia caesia* (L.) Maslin, Seigler & Ebinger	IC
	*Wisteriopsis reticulata* (Benth.) J. Compton & Schrire	RC
Gesneriaceae	*Aeschynanthus acuminatus* Wall. ex A. DC	FC
Heliotropiaceae	*Heliotropium sarmentosum* (Lam.) Craven	RC
Hernandiaceae	*Illigera luzonensis* (C. Presl) Merr	FC
Hydrangeaceae	*Hydrangea anomala* D. Don	RC
	^a^*Hydrangea fauriei* (Hayata) Y. De Smet & Granados	RC
	*Hydrangea integrifolia* Hayata	RC
	*Hydrangea viburnoides* (Hook. f. & Thomson) Y. De Smet & Granados var*. parviflora* Oliv. ex Maxim	RC
Lardizabalaceae	*Akebia longeracemosa* Matsum	VS
	*Akebia chingshuiensis* T. Shimizu	VS
	*Stauntonia hexaphylla* (Thunb.) Dcene	VS
	*Stauntonia obovata* Hemsl	VS
	^a^ *Stauntonia purpurea* Y. C. Liu & F. Y. Lu	VS
Loganiaceae	*Gardneria multiflora* Makino	FD, TR
	*Gardneria nutans* Siebold & Zucc	FD, TR
	*Strychnos cathayensis* Merr	TE, TR
Malpighiaceae	*Hiptage benghalensis* (L.) Kurz	RC
	*Tristellateia australasiae* A. Rich	FC
Melastomataceae	^a^*Medinilla formosana* Hayata	RC
	^a^*Medinilla hayataina* H. Keng	TR
Menispermaceae	*Cissampelos pareira* L. var. *hirsuta* (DC.) Forman	VS
	*Cocculus orbiculatus* (L.) DC	VS, SC
	^a^*Cyclea gracillima* Diels	VS
	*Cyclea insularis* (Makino) Hatus	VS
	^a^*Cyclea ochiaiana* (Yamam.) S. F. Huang & T. C. Huang	VS
	^a^*Paratinospora dentata *(Diels) Wei Wang	VS
	*Pericampylus glaucus* (Lam.) Merr	VS
	*Sinomenium acutum* (Thunb.) Rehder & E. H. Wils	VS
	*Stephania japonica* (Thunb.) Miers	VS
	*Stephania longa* Lour	VS
	*Stephania merrillii* Diels	VS
	*Stephania tetraandra* S. Moore	VS
	*Tinospora crispa* (L.) J. D. Hook. & Thom	VS
	*Tinospora sinensis* (Lour.) Merr	VS
Moraceae	*Ficus aurantiaca* Griff. var. *parvifolia* (Corner) Corner	IC
	*Ficus pumila* L. var. *awkeotsang* (Makino) Corner	IC
	*Ficus pumila* L. var. *pumila*	IC
	*Ficus sarmentosa* Buch.-Ham. ex Sm. var. *nipponica* (Franch. & Sav.) Corner	IC
	^a^*Ficus trichocarpa* Blume var. *obtusa* (Hassk.) Corner	IC
	*Ficus vaccinioides* Hemsl. ex King	RC
	*Maclura cochinchinensis* (Lour.) Corner	RC
	*Malaisia scandens* (Lour.) Planch	RC
Nyctaginaceae	*Bougainvillea spectabilis* Willdenow	SC
	*Pisonia aculeata* L	TE
Oleaceae	*Jasminum lanceolarium* Roxb	IC
	*Jasminum nervosum* Lour	RC
	*Jasminum sinense* Hemsl	RC
	*Jasminum urophyllum* Hemsl	RC
Opiliaceae	*Cansjera rheedei* J. F. Gmelin	RC
Pandanaceae	*Freycinetia formosana* Hemsl	RC
Passifloraceae	*Passiflora biflora* Lam	FC
	*Passiflora edulis* Sims	FC
	*Passiflora laurifolia* L	RC
	*Passiflora quadrangularis* L	IC
	*Passiflora suberosa* L. subsp. *litoralis* (Kunth) K.Port.-Utl. ex M.A.M.Azevedo, Baumbratz & Gonç.-Estev	FC
	*Passiflora vesicaria* L	FC
Phyllanthaceae	*Phyllanthus reticulatus* Poiret	FC
Piperaceae	*Piper arborescens* Roxb	EP, VS
	*Piper betle* L	EP, VS
	*Piper interruptum* Opiz	EP, VS
	*Piper kadsura* (Choisy) Ohwi	EP, VS
	^a^ *Piper kawakamii* Hayata	EP, VS
	^a^*Piper lanyuense* K.N. Kung & Kun C. Chang	EP, VS
	^a^*Piper sintenense* Hatusima	EP, VS
	^a^*Piper taiwanense* T.T. Lin & S.Y. Lu	EP, VS
Polygonaceae	*Antigonon leptopus* Hook. & Arn	SC
	*Persicaria chinense* (L.) H. Gross	VS
	*Persicaria perfoliata* (L.) H. Gross	FC
	*Reynoutria multiflorum* (Thunb.) Moldenke var. *hypoleuca* (Ohwi) S.S. Ying	VS
Primulaceae	^a^*Embelia laeta* (L.) Mez var. *papilligera* (Nakai) Walker	VS
	^a^*Embelia lenticellata* Hayata	VS
	*Embelia rudis* Hand.-Mazz	VS
Ranunculaceae	*Clematis chinensis* Osbeck var. *chiensis*	VS
	*Clematis crassifolia* Benth	VS
	^a^*Clematis formosana* Kuntz	VS
	^a^*Clematis gouriana* Roxb. *ex* DC. subsp. *lishanensis* Yang & Huang	VS, FD
	*Clematis grata* Wall	VS
	*Clematis henryi* Oliv. var. *henryi*	VS
	*Clematis lasiandra* Maxim	VS
	*Clematis leschenaultiana* DC	VS
	*Clematis meyeniana* Walp	VS
	*Clematis montana* Buch.-Ham. *ex* DC	VS
	^a^*Clematis parviloba* Gard. ex Champ. subsp*. bartlettii* (Yamamoto) Yang & Huang	VS
	*Clematis tamurae* T. Y. A. Yang & T. C. Huang	VS
	^a^*Clematis tashiroi* Maxim. var. *tashiroi*	VS
	*Clematis terniflora* DC. var. *garanbiensis* (Hayata) M. C. Chang	VS
	*Clematis uncinata* Champ. ex Benth. var. *okinawensis* (Ohwi) Ohwi	VS
	*Clematis uncinata* Champ. ex Benth.var. *uncinata*	VS
Rhamnaceae	^a^*Berchemia arisanensis* Y. C. Liu & F. Y. Lu	RC
	^a^*Berchemia fenchifuensis* C. M. Wang & F. Y. Lu	RC
	*Berchemia formosana* C. K. Schneid	RC
	*Berchemia racemosa* Siebold & Zucc. var. *magna* Makino	RC
	^a^*Rhamnus formosana* Matsum	RC
	^a^*Sageretia randaiensis* Hayata	RC
	*Sageretia thea* (Osbeck) M. C. Johnston var. *thea* (Osbeck) M. C. Johnston	RC
	^a^*Ventilago elegans* Hemsl	RC
	*Ventilago leiocarpa* Benth	RC
Rosaceae	*Rubus formosensis* Kuntze	RC
	*Rubus pyrifolius* Sm	RC
	*Rubus rolfei* S. Vidal	RC
	*Rubus swinhoei* Hance var. *swinhoei*	RC
	*Rubus wallichianus* Wight & Arn	IC
Rubiaceae	*Coptosapelta diffusa* (Champ. ex Benth.) Steenis	FC, IC
	*Dimetia hedyotidea* (DC.) T. C. Hsu	DX
	*Morinda parvifolia* Bartl	FC
	*Morinda umbellata* L	FC
	*Mussaenda formosanum* (Matsum.) T. Y. Aleck Yang & K. C. Huang	RC
	*Mussaenda parviflora* Miq	RC
	*Mussaenda pubescens* W. T. Aiton	RC
	^a^*Mussaenda taihokuensis* Masam	RC
	*Paederia cavaleriei* H. Lév	IC, VS
	*Paederia foetida* L	IC, VS
	*Psychotria serpens* L	RC
	*Randia sinensis* (Lour.) Roem. &Schult	RC
	*Uncaria hirsuta* Havil	RC
	*Uncaria lanosa* Wall. var. *appendiculata* Ridsdale	IC
	*Uncaria rhynchophylla* (Miq.) Miq. ex Havil	RC
Rutaceae	*Toddalia asiatica* (L.) Lam	RC
	*Zanthoxylum nitidum* (Roxb.) DC	RC
	*Zanthoxylum scandens* Blume	RC
Sabiaceae	*Sabia swinhoei* Hemsl	VS
	^a^*Sabia transarisanensis* Hayata	VS
Schisandraceae	*Kadsura japonica* (L.) Dunal	RC
	*Kadsura matsudae* Hayata	RC
	^a^*Schisandra arisanensis* Hayata	RC
Solanaceae	*Solanum lyratum* Thunb	TR
	*Solanum pittosporifolium* Hemsl	TR
Vitaceae	*Ampelopsis brevipedunculata* (Maxim.) Trautv. var. *ciliata* (Nakai) F. Y. Lu	IC
	*Ampelopsis brevipedunculata* (Maxim.) Trautv. var. *hancei* (Planch.) Rehder	IC
	*Cayratia corniculata* (Benth.) Gagnep	IC,VS, SC
	*Cayratia japonica* (Thunb.) Gagnep	IC,VS
	*Cayratia maritime* B. R. Jackes	VS
	*Cissus assamica* (Laws.) Craib	IC,VS
	^a^*Cissus pteroclada* Hayata	IC,VS
	*Cissus repens* Lam	IC,VS
	*Cissus* sp.	IC,VS
	*Cissus verticillata* (L.) Nicolson & C.E.Jarvis	IC,VS
	*Nekemias cantoniensis* (Hook. & Arn.) J.Wen & Z.L.Nie	VS
	*Nekemias cantoniensis* (Hook. & Arn.) J.Wen & Z.L.Nie var. *leecoides* (Maxim.) F.Y.Lu	VS
	*Parthenocissus tricuspidata* (Siebold & Zucc.) Planch	IC,VS
	^a^*Tetrastigma formosanum* (Hemsley) Gagnepain	IC,VS, SC
	^a^*Tetrastigma hemsleyanum* Diels & Gilg	IC,VS, SC
	^a^*Tetrastigma lanyuense* C. E. Chang	IC,VS, SC
	*Tetrastigma obtectum* (Wallich ex M. A. Lawson) Planchon ex Franchet var. *glabrum* (H. Léveillé) Gagnepain	IC,VS
	*Vitis amurenensis* Rupr	IC
	*Vitis flexuosa* Thunberg	IC
	^a^*Vitis heyneana* Roemer & Schultes	IC
	*Vitis heyneana* Roemer & Schultes subsp. *ficifolia* (Bunge) C. L. Li	IC
	*Vitis sinocinerea* W. T. Wang	IC
52 families	287 species	

RC: stem cambium normal in production and round in conformation; VS: axial vascular elements in segments; IC: stem cambium normal in production but stem with irregular conformation; TR: intraxylary phloem; FC: furrowed xylem of cambium continuity; SC: successive cambia; FD: furrowed xylem of cambium discontinuity; TE: interxylary phloem; EP: external primary vascular cylinders; ES: external secondary vascular cylinders; DX: fissured stem/dispersed xylem

^ * ^Endemic species

The cambial variants were divided into two categories, a single cambium and multiple cambia, as defined by Angyalossy et al. (2015). Single cambial variants were subdivided into five types, including irregular conformation, interxylary phloem, furrowed xylem, axial vascular elements in segments, and fissured stem/dispersed xylem, and are described below:

1. Irregular conformation (IC): Vascular cambium has a regular activity, the proportions of xylem and phloem produced varied around the girth, and the stem with irregular conformation can be divided into lobes and flattened.
2. Interxylary phloem (TE): Interxylary phloem is defined as strands or bands of phloem embedded within the secondary xylem of a stem of a plant that has a single vascular cambium (Carlquist 2001, 2013). Interxylary phloem have four different ontogenetic origins (Angyalossy et al. 2015), (a) the cambium produces phloem in both directions (inside and outside), followed by the formation of xylem only towards the inside, as a result, the phloem enclosd in the wood; (b) the cambium produces less xylem than phloem at the certain portions of stem, creating small phloem arcs, including strands (Caballé 1993), bands (Metcalfe and Chalk 1985) or umbrellas; (c) the result of the inclusion of phloem wedges within the xylem; and (d) the cambium varies from the differentiation of xylem parenchyma into sieve tubes. The evenly distributions or round islands of interxylary phloem generate patterns that are available in the identification of species.
3. Furrowed xylem (FX): This type of variant is derived from the part of the cambium that contains a relatively small amount of xylem and a relatively large amount of phloem; it is characterized by a regular gap between the xylem and phloem or irregular arrangements of the two. One of the furrowed xylem has a regular cambium, forms four equidistant phloem arcs/wedges, and then develops into a multiple of four phloem arcs/wedges (Angyalossy et al. 2012). This process result in multiple phloem arcs/wedges evenly spread across the stem. The other of the furrowed xylem is irregular spacing of phloem arcs/wedges. Caballé (1993) identified the furrowed xylem of four regular gaps as quarter-lobes. The furrowed xylem is divided into two groups according to the discontinuity (FD) or continuity (FC) of the cambium. Discontinuity cambium results in the inclusion of fragments of the original concentric cambium within the phloem wedges, which are radially lined by wide rays (Pace et al. 2009). A continuous cambium produces secondary phloem that contains tanniniferous cells and druse crystals, or the sclerenchyma is composed of large sclereid clusters or bands of fiber-sclereids to form a stratified phloem (Pace et al. 2009). The cambium continuity also can be divided into shallow or deep split types by the depth of the phloem wedges (Angyalossy et al. 2015).
4. Axial vascular elements in segments (VS): Axial elements of the xylem and phloem are present in segments alternating with very wide xylem and phloem rays, and also called as xylem in plates (Carquist 1991, 2001). Angyalossy et al. (2012) defined this feature as axial vascular elements in segments.
5. Fissured stem/dispersed xylem (DX): this cambial variant is defined as: irregularly shaped, disoriented strands of xylem and phloem associated with fragments of vascular cambium spread throughout a parenchymatous matrix; or the dispersed nature of xylem and phloem strands results from the rupturing of the vascular cambium by parenchyma cells (Ayensu and Stern 1964; Cabanillas et al. 2017). This cambial variant type is derived from non-lignified parenchyma proliferation and is called dispersed xylem segments, or is more cracked than the normal xylem, also known as fissured/blocks xylem. The primary xylem is severed by the proliferation of phloem or parenchyma cells (Metcalfe and Chalk 1985; Carlquist 2001).

Multiple cambia are derived from the parenchyma of the cortex in order to generate lateral meristems, which begin to produce conjunctive tissue and vascular bundle cambium inward, and the epidermis outward. The cambial variants of this category can be divided into four types:

1. Successive cambia (SC): Successive cambia are formed from new cambia arising through cell division in the external secondary vascular system. Each new cambia in the stem successively generates a secondary inward xylem and a secondary outward phloem. The process involves pericyclic, cortex, and parenchyma cell division. Pericyclic or cortical cell division causes a meristematic zone, which differentiates into parenchyma or sclerenchyma, called connective tissue. The successive cambia are formed by alternating concentric rings of xylem and phloem. The xylem and phloem produced by the cambium are in their normal positions (Metcalfe and Chalk 1985), and vascular tissue is separated by a band of parenchyma or sclerenchyma (conjunctive tissue). The continuous cambium is also referred to as the alternation of bands of vascular tissue with connective tissue (Acevedo-Rodríguez 2005). The successive cambia are further subdivided into two types, concentric and non-concentric bands. The pith of concentric and non-concentric bands is usually located in the center or eccentric of these rings (Isnard and Silk 2009). However, the pith in this variety is not located in the center of the rings and is defined as successive and directional cambia (Isnard and Silk 2009).
2. Intraxylary phloem (TR): Intraxylary phloem is derived from the formation of a secondary cambium between the primary xylem and the pith (Angyalossy et al. 2015). The secondary cambium either produces a phloem ring that appears inside the xylem, or two arcs of phloem toward the pith (Metcalfe and Chalk 1985). Intraxylary phloem refer to phloem strands that occur adjacent to protoxylem, at margins of the pith (Carlquist 2013). Internal phloem had been used to refer to intraxylary phloem, but it is vague with respect to ontogeny as well as location of phloem. Due to conflicting usages, internal phloem had been rejected.
3. External primary vascular cylinders (EP): This type is formed by the secondary growth of external primary vascular bundles, such as in Piperaceae.
4. External secondary vascular cylinders (ES): This type is developed from the neoformation of secondary vascular cylinders. The cambium of the cortex is formed and developed into vascular bundles cylinders of different diameters, which surround the primary vascular bundle cylinders creating external secondary vascular cylinders (Angyalossy et al. 2012, 2015).

Other common traits of lianas, such as a single species exhibiting more than one type of cambial variant or the parenchyma proliferation of the stem association with one type of cambial variant (Angyalossy et al. 2015), should be investigated. Therefore, we recorded the combination type and parenchymatization in our samples. The features of bark and cross-section of the stem were described based on definitions provided by Yang et al. (2021).

## Results and discussion

### Patterns of cambial variants of Taiwan lianas

**Fig. 1:**
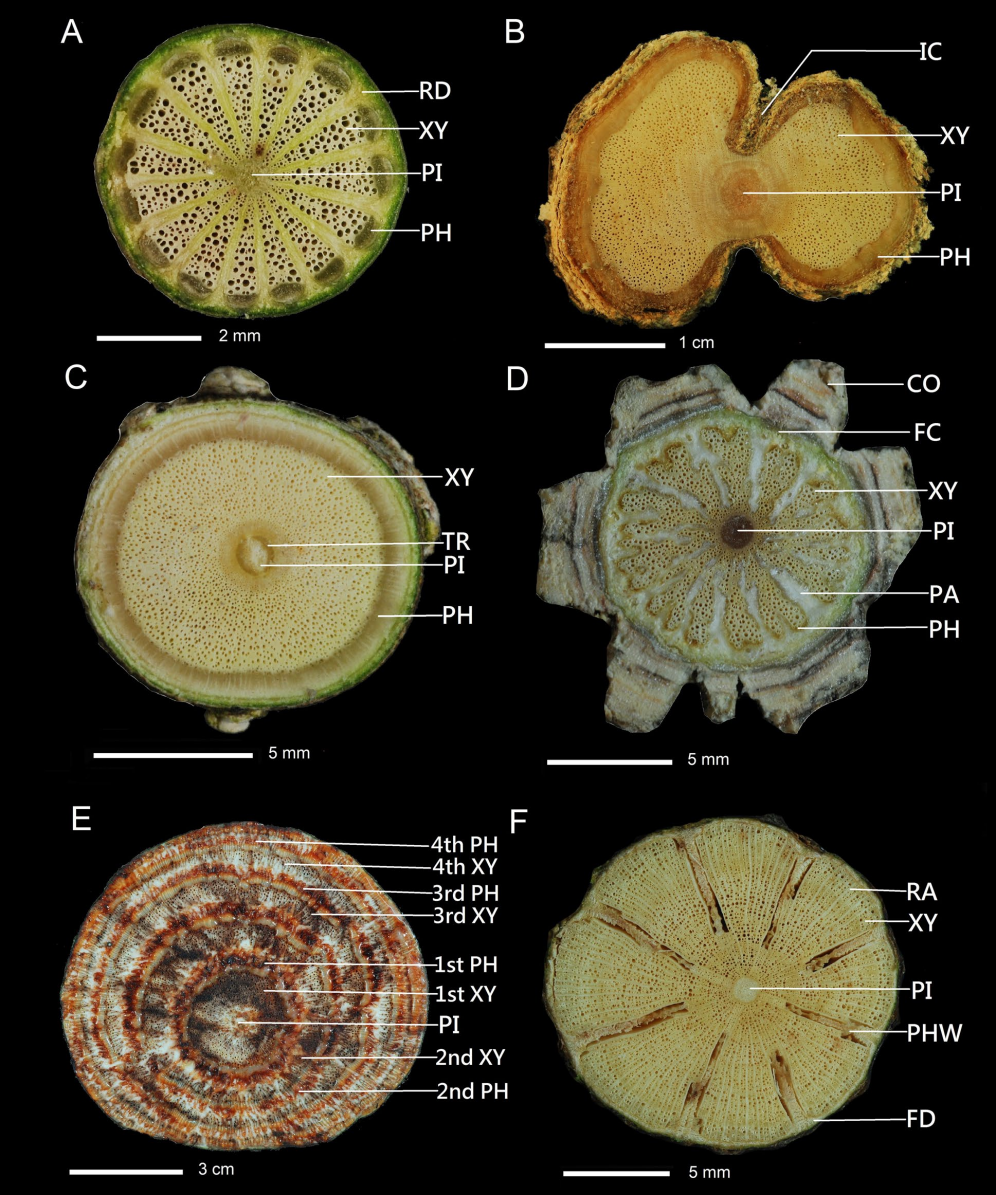
Single cambial variant of stem cross section. **A** Axial vascular elements in segments: *Pericampylus formosanus.* **B** Irregular in conformation: *Jasminum lanceolarium*. **C** Intraxylary phloem: *Melodinus  angustifolius*. **D** Furrowed xylem of cambium continuity: *Morinda parvifolia*. **E** Successive cambia: *Mucuna macrocarpa*. **F** Furrowed xylem of cambium discontinuity: *Pyrostegia venusta*. RD, ray dilatation, PI, pith, XY, xylem, PH, phloem, IC, irregular conformation, TR, intraxylary phloem, CO, cork, FC, furrowed xylem of cambium continuity, PA, parenchyma, 1st XY, first xylem, 2nd XP, second xylem, 3rd XP, third xylem, 4th XY, forth xylem, 1st PH, first phloem, 2nd PH, second phloem, 3rd PH, third phloem, 4th PH, forth phloem, FD, furrowed xylem of cambium discontinuity, PHW, phloem wedges-like

**Fig. 2:**
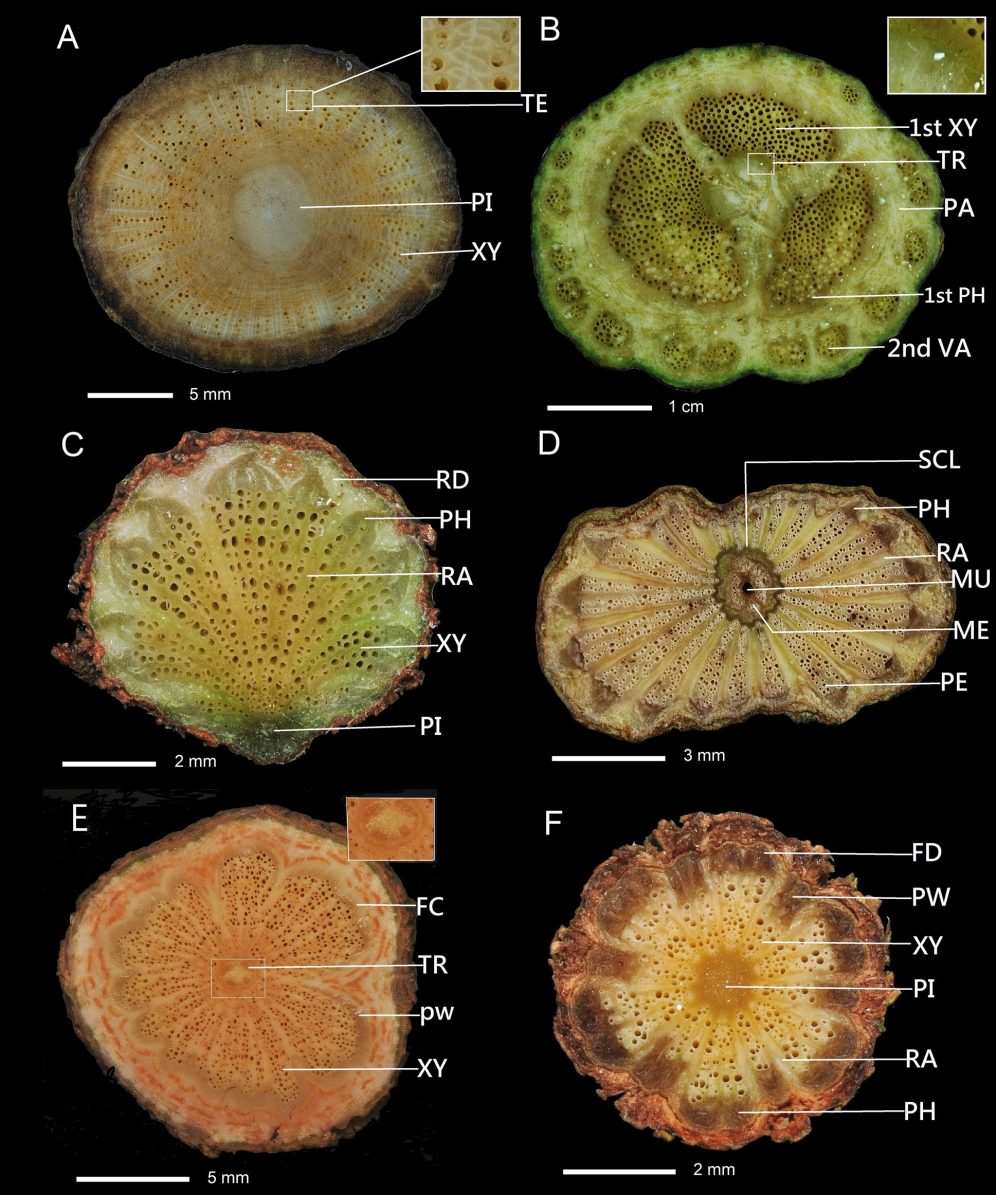
Single cambial variant or combination of two cambial variants of stem cross section. **A** Interxylary phloem (enlargement): *Thunbergia grandiflora*. **B** Intraxylary phloem (enlargement) combined with successive cambia: *Distimake tuberosus*. **C** Irregular conformation combined with axial vascular elements in segments: *Tetrastigma obtectum* var. *glabrum.* **D** External primary vascular cylinders combined with axial vascular elements in segments: *Piper kadsura*. **E** Furrowed xylem of cambium continuity combined with intraxylary phloem: *Gymnema sylvestre*. **F** Furrowed xylem of cambium discontinuity combined with axial vascular elements in segments: *Clematis gouriana* subsp. *lishanensis*. PI, pith, TE, interxylary phloem, TR, intraxylary phloem, 1st XY, first xylem, 2nd XP, second xylem, 1st PH, first phloem, RA, ray, XY, xylem, PH, phloem, SCL, sclenchyma, MU, mucilage canal, ME, medullary vascular bundles, PE, peripheral vascular bundles, FD, furrowed xylem of cambium discontinuity, FC, furrowed xylem of cambium continuity, PW, phloem wedges

Among the 287 species (Table 1) of lianas in this study, 96 species had regular cambium development with a circular cross-section, and 191 species had other cambial variants (Table 2). Among the 191 species, eight cambial variants were exhibited in 128 species, and fourteen combinations of cambial variants were present in 63 species. Furthermore, of these 191 species, 50 had axial vascular elements in segments, e.g., *Pericampylus formosanus* (Fig. 1A), 23 species had irregular conformation, e.g., *Jasminum lanceolarium* (Fig. 1B), 21 species had intraxylary phloem, e.g., *Melodinus  angustifolius* (Fig. 1C), 17 species had furrowed xylem of cambium continuity, e.g., *Morinda parvifolia* (Fig. 1D), 10 species had successive cambia, e.g., *Mucuna macrocarpa* (Fig. 1E), three species had furrowed xylem of cambium discontinuity, e.g., *Pyrostegia venusta* (Fig. 1F), and three species had interxylary phloem, e.g., *Thunbergia grandiflora* (Fig. 2A), one species had fissured stem/dispersed xylem, e.g., *Dimetia hedyotidea* (Fig. 4D).

**Table 2:** Cambial variant types of lianas of different families in Taiwan

Family	RC	VS	IC	TR	FC	SC	FD	TE	DX	TR + SC	IC + VS	EP + VS	FC + TR	FD + VS
Acanthaceae	–	–	–	–	1	–	–	2	–	–	–	–	–	–
Actinidiaceae	5	–	–	–	–	–	–	–	–	–	–	–	–	–
Amaranthaceae	–	–	–	–	–	1	–	–	–	–	–	–	–	–
Anacardiaceae	1	–	–	–	–	–	–	–	–	–	–	–	–	–
Annonaceae	3	–	–	–	–	–	–	–	–	–	–	–	–	–
Apocynaceae	–	–	–	14	–	–	–	–	–	–	–	–	3	–
Araliaceae	3	–	–	–	–	–	–	–	–	–	–	–	–	–
Areacaceae	2	–	–	–	–	–	–	–	–	–	–	–	–	–
Aristolochiaceae	–	4	–	–	–	–	–	–	–	–	–	–	–	–
Asteraceae	1	2	1	–	–	–	1	–	–	–	–	–	–	–
Basellaceae	–	–	–	–	–	–	–	–	–	–	–	–	–	–
Bignoniaceae	–	–	–	–	–	–	2	–	–	–	–	–	–	–
Cannabaceae	–	–	1	–	–	–	–	–	–	–	–	–	–	–
Capparaceae	2	–	–	–	–	–	–	–	–	–	–	–	–	–
Caprifoliaceae	3	–	–	–	–	–	–	–	–	–	–	–	–	–
Cecropiaceae	1	–	–	–	–	–	–	–	–	–	–	–	–	–
Celastraceae	2	1	1	–	2	–	–	–	–	–	–	–	–	–
Combretaceae	–	–	–	–	–	–	–	–	–	–	–	–	1	–
Connaraceae	–	–	–	–	–	1	–	–	–	–	–	–	–	–
Convolvulaceae	–	–	–	4	–	–	–	–	–	20	–	–	–	–
Cucurbitaceae	–	–	–	–	–	–	–	–	–	–	3	–	–	1
Elaeagnaceae	5	–	–	–	–	–	–	–	–	–	–	–	–	–
Euphorbiaceae	–	–	1	–	–	–	–	–	–	–	–	–	–	–
Fabaceae	24	–	4	–	3	6	–	–	–	–	–	–	–	–
Gesneriaceae	–	–	–	–	1	–	–	–	–	–	–	–	–	–
Heliotropiaceae	1	–	–	–	–	–	–	–	–	–	–	–	–	–
Hernandiaceae	–	–	–	–	1	–	–	–	–	–	–	–	–	–
Hydrangeaceae	4	–	–	–	–	–	–	–	–	–	–	–	–	–
Lardizabalaceae	–	5	–	–	–	–	–	–	–	–	–	–	–	–
Loganiaceae	–	–	–	–	–	–	–	–	–	–	–	–	–	–
Malpighiaceae	1	–	–	–	1	–	–	–	–	–	–	–	–	–
Melastomataceae	1	–	–	1	–	–	–	–	–	–	–	–	–	–
Menispermaceae	–	13	–	–	–	–	–	–	–	–	–	–	–	–
Moraceae	4	–	4	–	–	–	–	–	–	–	–	–	–	–
Nyctaginaceae	–	–	–	–	–	1	–	1	–	–	–	–	–	–
Oleaceae	3	–	1	–	–	–	–	–	–	–	–	–	–	–
Opiliaceae	1	–	–	–	–	–	–	–	–	–	–	–	–	–
Pandanaceae	1	–	–	–	–	–	–	–	–	–	–	–	–	–
Passifloraceae	1	–	1	–	4	–	–	–	–	–	–	–	–	–
Phyllanthaceae	–	–	–	–	1	–	–	–	–	–	–	–	–	–
Piperaceae	–	–	–	–	–	–	–	–	–	–	–	8	–	–
Polygonaceae	–	2	–	–	1	1	–	–	–	–	–	–	–	–
Primulaceae	–	3	–	–	–	–	–	–	–	–	–	–	–	–
Ranunculaceae	–	15	–	–	–	–	–	–	–	–	–	–	–	1
Rhamnaceae	9	–	–	–	–	–	–	–	–	–	–	–	–	–
Rosaceae	4	–	1	–	–	–	–	–	–	–	–	–	–	–
Rubiaceae	8	–	1	–	2	–	–	–	1	–	2	–	–	–
Rutaceae	3	–	–	–	–	–	–	–	–	–	–	–	–	–
Sabiaceae	–	2	–	–	–	–	–	–	–	–	–	–	–	–
Schisandraceae	3	–	–	–	–	–	–	–	–	–	–	–	–	–
Solanaceae	–	–	–	2	–	–	–	–	–	–	–	–	–	–
Vitaceae	–	3	7	–	–	–	–	–	–	–	8	–	–	–
Sum of family	25	11	11	4	10	5	2	2	1	1	3	1	2	2
Sum of species	96	50	23	21	17	10	3	3	1	20	13	8	4	2
Percentage %	33.4	17.4	8.0	7.3	5.5	3.5	1.0	1.0	0.3	7.0	4.5	2.8	1.4	0.7

Family	FD + TR	SC + VS	FC + IC	ES + TR	TE + TR	ES + IC	IC + DX	IC + VS + SC	FD + VS + SC	SP No.	Types No.
Acanthaceae	–	–	–	–	–	–	–	–	–	3	2
Actinidiaceae	–	–	–	–	–	–	–	–	–	5	0
Amaranthaceae	–	–	–	–	–	–	–	–	–	1	1
Anacardiaceae	–	–	–	–	–	–	–	–	–	1	0
Annonaceae	–	–	–	–	–	–	–	–	–	3	0
Apocynaceae	–	–	–	1	–	–	–	–	–	18	3
Araliaceae	–	–	–	–	–	–	–	–	–	3	0
Areacaceae	–	–	–	–	–	–	–	–	–	2	0
Aristolochiaceae	–	–	–	–	–	–	–	–	–	4	1
Asteraceae	–	–	–	–	–	–	–	–	–	5	3
Basellaceae	–	1	–	–	–	–	–	–	–	1	1
Bignoniaceae	–	–	–	–	–	–	–	–	–	2	1
Cannabaceae	–	–	–	–	–	–	–	–	–	1	1
Capparaceae	–	–	–	–	–	–	–	–	–	2	0
Caprifoliaceae	–	–	–	–	–	–	–	–	–	3	0
Cecropiaceae	–	–	–	–	–	–	–	–	–	1	0
Celastraceae	–	–	–	–	–	–	–	–	–	6	3
Combretaceae	–	–	–	–	–	–	–	–	–	1	1
Connaraceae	–	–	–	–	–	–	–	–	–	1	1
Convolvulaceae	–	–	–	–	–	–	–	–	–	24	2
Cucurbitaceae	–	–	–	–	–	–	–	2	1	7	4
Elaeagnaceae	–	–	–	–	–	–	–	–	–	5	0
Euphorbiaceae	–	–	–	–	–	–	–	–	–	1	1
Fabaceae	–	–	–	–	–	1	1	–	–	39	5
Gesneriaceae	–	–	–	–	–	–	–	–	–	1	1
Heliotropiaceae	–	–	–	–	–	–	–	–	–	1	0
Hernandiaceae	–	–	–	–	–	–	–	–	–	1	1
Hydrangeaceae	–	–	–	–	–	–	–	–	–	4	0
Lardizabalaceae	–	–	–	–	–	–	–	–	–	5	1
Loganiaceae	2	–	–	–	1	–	–	–	–	3	2
Malpighiaceae	–	–	–	–	–	–	–	–	–	2	1
Melastomataceae	–	–	–	–	–	–	–	–	–	2	1
Menispermaceae	–	1	–	–	–	–	–	–	–	14	1
Moraceae	–	–	–	–	–	–	–	–	–	8	1
Nyctaginaceae	–	–	–	–	–	–	–	–	–	2	2
Oleaceae	–	–	–	–	–	–	–	–	–	4	1
Opiliaceae	–	–	–	–	–	–	–	–	–	1	0
Pandanaceae	–	–	–	–	–	–	–	–	–	1	0
Passifloraceae	–	–	–	–	–	–	–	–	–	6	3
Phyllanthaceae	–	–	–	–	–	–	–	–	–	1	1
Piperaceae	–	–	–	–	–	–	–	–	–	8	1
Polygonaceae	–	–	–	–	–	–	–	–	–	4	3
Primulaceae	–	–	–	–	–	–	–	–	–	3	1
Ranunculaceae	–	–	–	–	–	–	–	–	–	16	2
Rhamnaceae	–	–	–	–	–	–	–	–	–	9	0
Rosaceae	–	–	–	–	–	–	–	–	–	5	1
Rubiaceae	–	–	1	–	–	–	–	–	–	15	5
Rutaceae	–	–	–	–	–	–	–	–	–	3	0
Sabiaceae	–	–	–	–	–	–	–	–	–	2	1
Schisandraceae	–	–	–	–	–	–	–	–	–	3	0
Solanaceae	–	–	–	–	–	–	–	–	–	2	1
Vitaceae	–	–	–	–	–	–	–	4	–	22	4
Sum of family	1	2	1	1	1	1	1	2	1		
Sum of species	2	2	1	1	1	1	1	6	1	287	
Percentage %	0.7	0.7	0.3	0.3	0.3	0.3	0.3	2.1	0.3	100	

RC, stem cambium normal in production and round in conformation; VS, axial vascular elements in segments; IC, stem cambium normal in production but stem with irregular conformation; TR, intraxylary phloem; FC, furrowed xylem of cambium continuity; SC, successive cambia; FD, furrowed xylem of cambium discontinuity; TE, interxylary phloem; EP, external primary vascular cylinders; ES, external secondary vascular cylinders; DX, fissured stem/dispersed xylem; SP No., species number; Types No., cambium variant types in each family, RC not included

**Fig. 3:**
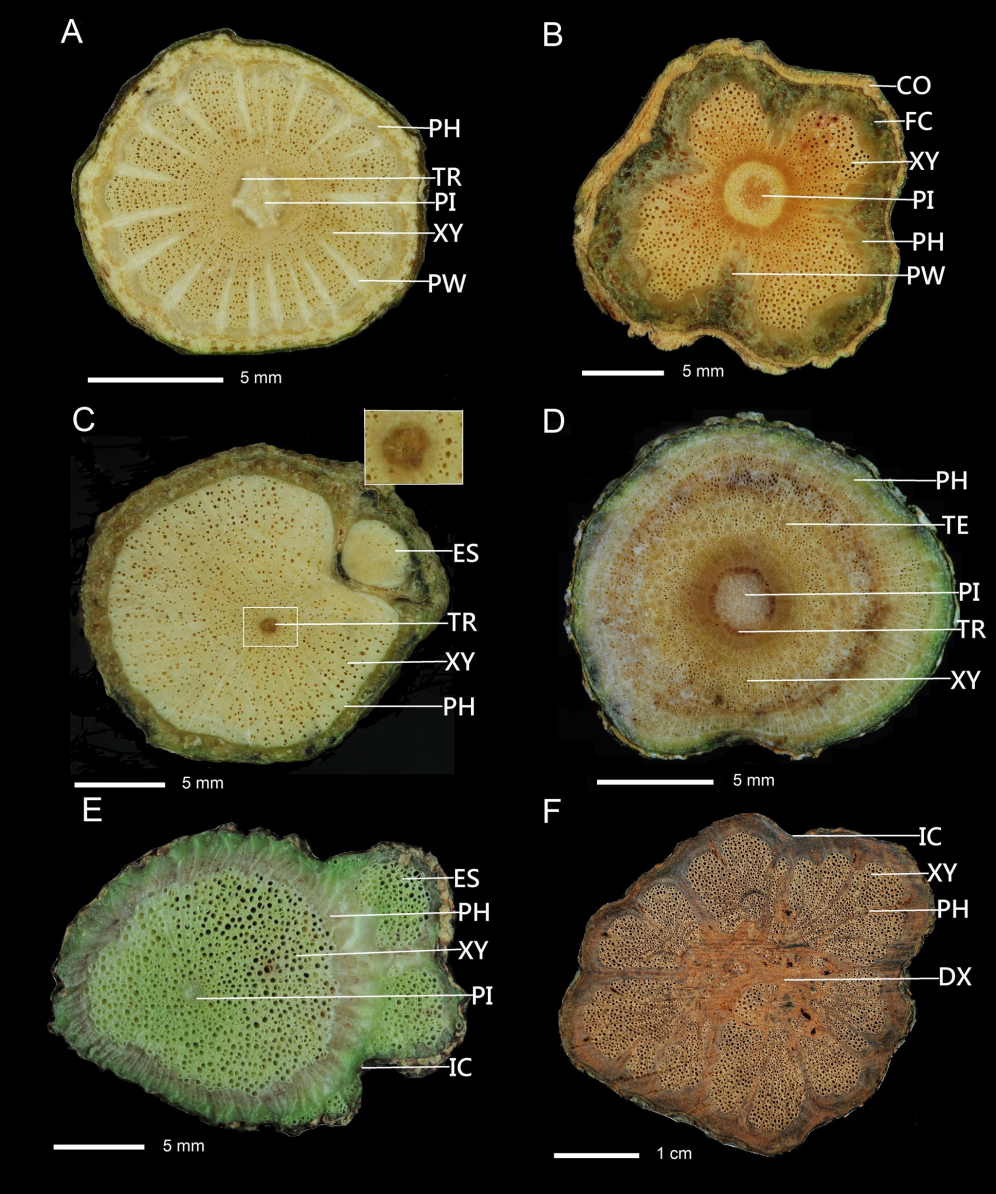
Combination of two cambial variants of stem cross section. **A** Furrowed xylem of cambium discontinuity combined with intraxylary phloem: *Gardneria multiflora*. **B** Furrowed xylem of cambium continuity combined with irregular conformation; *Coptosapelta diffusa.* **C** External secondary vascular cylinders combined with intraxylary phloem: *Trachelospermum gracilipes.* **D** Interxylary phloem combined with intraxylary phloem: *Strychnos cathayensis*. **E** External secondary vascular cylinders combined with irregular conformation: *Lablab purpureus.* **F** Fissured stem/dispersed xylem combined with irregular conformation: *Bauhinia championii*. PI, pith, FC, furrowed xylem of cambium continuity, PW, phloem wedges, IC, irregular conformation, PA, parenchyma, DX, fissured stem/ dispersed xylem, TR, intraxylary phloem, ES, external secondary vascular cylinders, XY, xylem, PH, phloem, TE, interxylary phloem

**Fig. 4:**
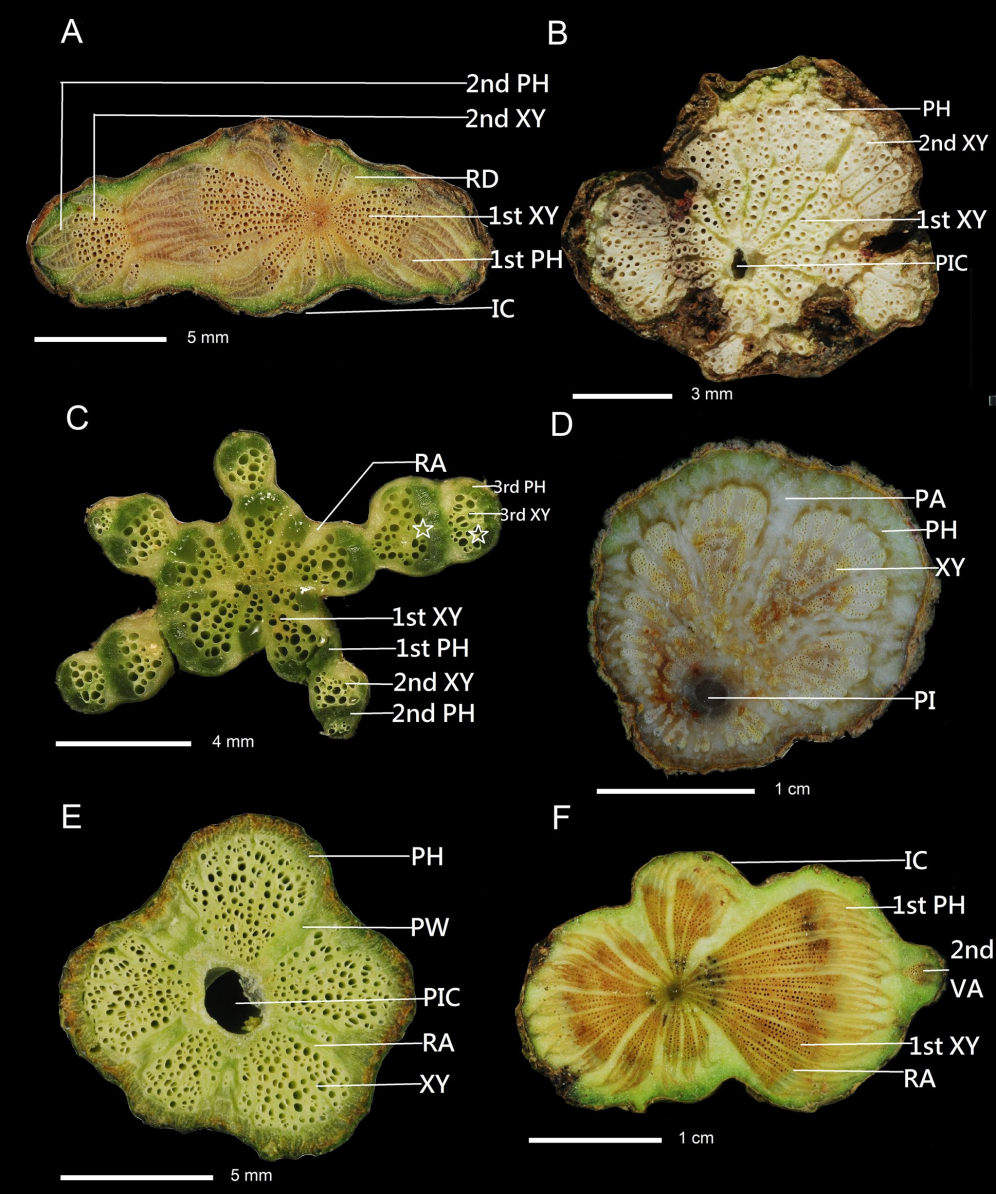
Combination of three cambial variants or single cambial variant of stem cross section. **A** Successive cambia combined with axial vascular elements in segments and irregular conformation: *Tetrastigma formosanum.* **B** Axial vascular elements in segments combined with successive cambia: *Anredera cordifolia.* **C** External secondary vascular cylinders (empty star) combined with axial vascular elements in segments and successive cambia: *Momordica charantia* var. *abbreviata*. **D** Fissured stem/dispersed xylem combined: *Dinetia hedyotidea.* **E** Furrowed xylem of cambium continuity: *Passiflora edulis.* **F** Irregular in conformation combined with axial vascular elements in segments and successive cambia: *Cayratia corniculata.* PI, pith, PIC, pith cavity, RA, ray, PW, phloem wedges, 1st XY, first xylem, 2nd XY, second xylem, 3rd XY, third xylem, 1st PH, first phloem, 2nd PH, second phloem, 3rd PH, third phloem, 2nd VA, second vascular bundle, IC, irregular conformation, PA, parenchyma proliferation, DX, fissured stem/dispersed xylem

Fourteen combination types of cambial variants were present, of which two or three combinations of cambial variants were present in 63 species. The two cambial variants combination included: 20 species having intraxylary phloem combined with successive cambia, e.g., *Distimake tuberosus* (Fig. 2B), 13 species with irregular conformation combined with axial vascular elements in segments, e.g., *Tetrastigma obtectum* var. *glabrum* (Fig. 2C), eight species having external primary vascular cylinders combined with axial vascular elements in segments, e.g., *Piper kadsura* (Fig. 2D), four species having intraxylary phloem combined with furrowed xylem of cambium continuity, e.g., *Gymnema sylvestre* (Fig. 2E), two species with axial vascular elements in segments combined with furrowed xylem of cambium discontinuity, e.g., *Clematis gouriana* subsp. *lishanensis* (Fig. 2F), two species possessing intraxylary phloem combined with furrowed xylem of cambium discontinuity, e.g., *Gardneria multiflora* (Fig. 3A), two species with axial vascular elements in segments combined with successive cambia, e.g., *Anredera cordifolia* (Fig. 4B) and *Cocculus orbiculatus*, one species with furrowed xylem of cambium continuity combined with irregular conformation, e.g., *Coptosapelta diffusa* (Fig. 3B), one species having external secondary vascular cylinders combined with intraxylary phloem, e.g., *Trachelospermum gracilipes* (Fig. 3C), one species possessing an interxylary phloem combined with intraxylary phloem, e.g., *Strychnos cathayensis* (Fig. 3D), one species having external secondary vascular cylinders with irregular conformation, e.g., *Lablab purpureus* (Fig. 3E), and one species having fissured stem/dispersed xylem combined with irregular conformation, e.g., *Bauhinia championii* (Fig. 3F).

The three cambial variants combination included: six species having successive cambia combined with axial vascular elements in segments and irregular conformation, e.g., *Tetrastigma formosanum* (Fig. 4A), *Momordica charantia* var. *abbreviata* (Fig. 4C), *Cayratia corniculata* (Fig. 4F), one species having furrowed xylem of cambium discontinuity combined with axial vascular elements in segments and successive cambia, such as *Momordica cochinchinensis*.

Axial vascular elements in segments type and intraxylary phloem type appeared in six and five combination types, respectively. These two variant types always appear in many combined variants, so they are more helpful to the lianas identification. Fourteen combinations types of cambial variants were found in Taiwan lianas, indicating multiple cambial variants type were found and could be a character of taxonomic values. The number and types of cambial variants in one family or genus might be related to the number of investigated species. Among the 52 families, most had only one type of variant. In this work Fabaceae and Rubiaceae showed to have the greatest diversity of cambial variants with five CV each one, followed by Cucurbitaceae and Vitaceae with four types. Other families like Apocynaceae, Asteraceae, Celastraceae, Passifloraceae, and Polygonaceae, had only three types (Table 3).

**Table 3:** List of orders and families of Taiwan lianas that possess the cambial variants

Orders	Families	Cambial variants (this study)	Angyalossy et al. (2015)
Asterales	Asteraceae	VS, FC, FD	VS, FX
Apiales	Araliaceae	RC	–
Arecales	Areacaceae	RC	–
Austrobaileyales	Schisandraceae	RC	–
Boraginales	Heliotropiaceae	RC	–
Brassicales	Capparaceae	RC	–
Caryophyllales	Amaranthaceae	SC	SC
	Nyctaginaceae	TE, SC	SC
	Polygonaceae	SC, VS, FC	IC, SC, TR
	Basellaceae	VS, TR, SC	–
Cornales	Hydrangeaceae	RC	–
Celastrales	Celastraceae	FC, VS, IC	FX, IC, SC,TE
Cucurbitales	Cucurbitaceae	IC + VS, VS + IS + SC, FD + VS, FD + VS + SC	SC, TE, VS
Dipsacales	Caprifoliaceae	RC	–
Ericales	Actinidiaceae	RC	–
	Primulaceae	VS	–
Fabales	Fabaceae	IC, DX + IC, FC, SC, ES	SC, TE, IC, FX, DX, TR
Gentianales	Apocynaceae	TR, TR + FC, TR + ES	IC, SC, TE, VS, FX
	Loganiaceae	TR + FD, TR + TE	TE
	Rubiaceae	FC + IC, IC, DX + TR + IC, IC + VS	FX, IC
Lamiales	Acanthaceae	FD, TE	SC, TE, DX
	Bignoniaceae	FD	FX, DX
	Gesneriaceae	FC	–
	Oleaceae	IC	–
Laurales	Hernandiaceae	FC	–
Magnoliales	Annonaceae	RC	–
Malpighiales	Euphorbiaceae	IC	ES
	Malpighiaceae	FC	FX, DX, SC, TE, IC
	Passifloraceae	FC	SC, FX
	Phyllanthaceae	FC	–
Myrtales	Combretaceae	TR + FC	TR, TE, ES, IC
Oxalidales	Connaraceae	SC	SC
Pandanales	Pandanaceae	RC	–
Piperales	Aristolochiaceae	VS	VS, IC
	Piperaceae	EP + VS	VS, IC, EP
Proteales	Sabiaceae	VS	–
Ranunculales	Menispermaceae	VS, VS + SC	SC, VS, IC, TR
	Ranunculaceae	VS, VS + FD	VS
	Lardizabalaceae	VS	–
Rosales	Cannabaceae	IC	–
	Cecropiaceae	RC	–
	Elaeagnaceae	RC	–
	Rhamnaceae	RC	–
	Rosaceae	RC	–
	Moraceae	IC	IC
Santalales	Opiliaceae	RC	–
Solanales	Convolvulaceae	TR, TR + SC	SC, TE, FX, DX, TR
	Solanaceae	TR	–
Vitales	Vitaceae	IC, VS, IC + VS + SC, VS + IC	VS, IC, SC, DX
18 orders	49 families		

RC, stem cambium normal in production and round in conformation; VS, axial vascular elements in segments; IC, stem cambium normal in production but stem with irregular conformation; TR, intraxylary phloem; FC, furrowed xylem of cambium continuity; SC, successive cambia; FD, furrowed xylem of cambium discontinuity; TE, interxylary phloem; EP, external primary vascular cylinders; ES, external secondary vascular cylinders; DX, fissured stem/dispersed xylem

Ten species belonging to Amaranthaceae, Connaraceae, Fabaceae, Nyctaginaceae, and Polygonaceae displayed successive cambia. In Fabaceae family, *Mucuna macrocarpa, Pueraria lobata* subsp. *thomsonii,* and *Pueraria montana* (Fig. 1E), have 3–4 layers of regular concentric rings. *Entada  phaseoloides* subsp. *phaseoloides*, *Entada phaseoloides* subsp. *tonkinensis,* and *Entada rheedei* also have continuous cambium, but their stem cross-section consists of 5–12 layers of irregular concentric rings. The species *Bougainvillea spectabilis* (Nyctaginaceae) also had successive cambia with 5–6 layers of regular concentric rings.

### Cambial variants of Taiwan lianas in each family or genus

The number of cambial variants in one family or genus might be related to the number of species investigated. Therefore, a family or genus with numerous species with only a single or few cambial variants may be easily identified using the unique cambial variants. For example, 14 out of 18 species of Apocynaceae possess intraxylary phloem; 19 out of 23 species of Convolvulaceae have a combination of intraxylary phloem and successive cambia; Lardizabalaceae (Yang et al. 2019), Menispermaceae (Yang et al. 2016), and Ranunculaceae (Yang et al. 2021), and 35 other species have axial vascular elements in segments; eight species of Piperaceae have a combination of external primary vascular bundle cylinder and axial vascular elements in segments (Yang and Chen 2017); 4 out of 6 species of *Passiflora* genus possess furrowed xylem; 12 out of 20 species of Vitaceae had a combination of axial vascular elements in segments and irregular in conformation.

Carquist (1999) indicated *Anredera baselloides* (Basellaceae) have combinations of three cambial variants, viz. successive cambia, interxylary phloem strands, and intraxylary phloem in a wider stem, with other features, such as restriction of vessels to central portions of fascicular areas, crystals and mucilage cells in cortex, and rays. We observed that the species *Anredera cordifolia* had axial vascular elements in segments in narrow stems and 2–3 layers of successive cambia in wider stems (Fig. 4B).

In this study, two species of Bignoniaceae had furrowed xylem of cambium discontinuity. Furrowed xylem of cambium discontinuity had two patterns. One where wedge-shaped phloem has a regular spacing, as found in *Pyrostegia venusta* (Bignoniaceae) (Fig. 1F) and *Clematis gouriana* subsp. *lishanensis* (Ranunculaceae) (Fig. 2F); the other had wedge-shaped phloem with irregular spacing, as in *Passiflora edulis* (Passifloraceae) (Fig. 4E). Furrowed xylem of cambium continuity had shallowly or deeply lobed patterns based on the depth of phloem arcs/wedges, as in *Morinda parvifolia* (Rubiaceae) (Fig. 1D) where the stem is deeply lobed, whereas *Gymnema sylvestre* (Apocynaceae) is shallowly lobed (Fig. 2E). However, four equidistant phloem arcs/wedges, a multiple of four phloem wedges (Angyalossy et al. 2015), cambium continuity/discontinuity, and the depth of phloem arcs/wedges in Bignoniaceae (Angyalossy et al. 2012) are the diagnostic characteristics available to the identification of furrowed xylem types. Although Angyalossy et al. (2012, 2015) distinguish some members of the subfamily Bignoniae, but phloem wedges are present in more families where they have a diversity of patterns and variability of conformation (Cabanillas et al. 2017; Quintanar and Pace 2022). So, studying the variability of phloem arcs/wedges in Taiwan lianas become more important.

**Fig. 5:**
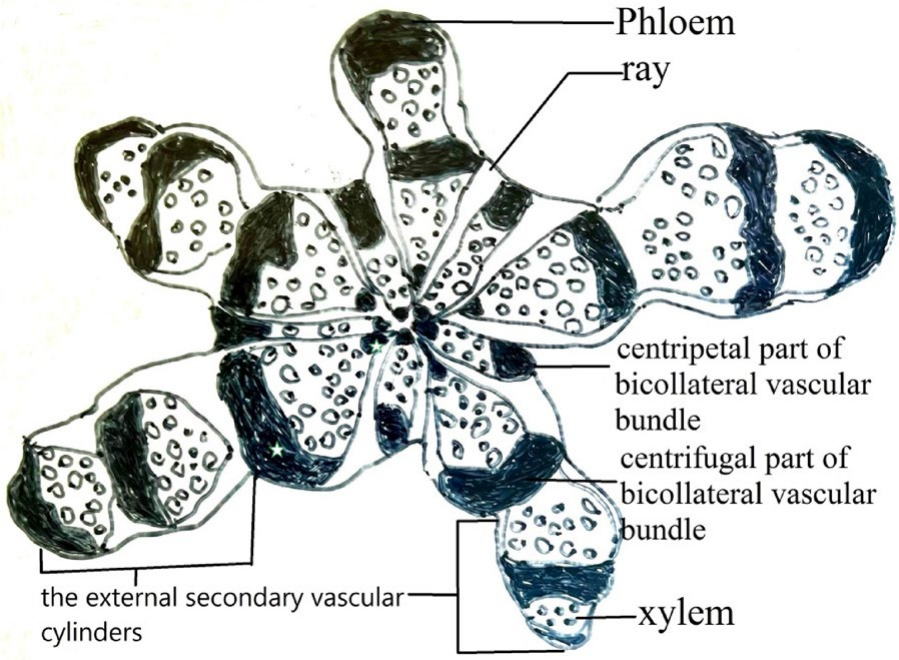
The bicollateral vascular bundle (Cucurbitaceae) with outer and inner phloem at both ends. White stars are inner and outer phloem

The diagnostic feature of the bicollateral vascular bundle (Cucurbitaceae) had outer and inner phloem at both ends (Figs. 4C, 5). The distribution of the bicollateral vascular bundle on the stem cross-section had centripetal and centrifugal parts. The centripetal part of bicollateral vascular bundle was composed of centripetal phloem and centrifugal xylem, and no cambium was found between them. In contrast, the centrifugal part has centripetal xylem, a cambium, and centrifugal phloem. The wider primary rays split the bicollateral vascular bundle, secondary rays appeared in the xylem, and the larger the diameter, the secondary rays were produced. The interfascicular cambium was located between the vascular bundles and contributed to radial growth (Schweingruber et al. 2011). However, the cortical bicollateral vascular bundle of *Momordica charantia* var. *abbreviata* developed one to five layers of centrifugal vascular bundles, forming directional continuous successive cambia. This species exhibited a combination of three cambial variants (Fig. 4C) different from the other species of Cucurbitaceae. The cambial variants of *Momordica cochinchinensis* and *Neoalsomitra integrifolia* had furrowed xylem of cambium discontinuity combined with axial vascular elements in segments, which were special and different from other species of Cucurbitaceae.

The cambial variants of *Passiflora edulis* (Passifloraceae) had furrowed xylem of cambium continuity formed from very shallow depressions, via shallow to deep phloem wedges (Rajput and Baijnath 2016). The other diagnostic feature of *P. edulis* was the presence of five pairs of interfascicular rays distributed equidistantly. The cambial variant of four *Passiflora* species in this study exhibited furrowed xylem of cambium continuity, but the cambia of *Passiflora laurifolia* had regular secondary growth and the stem was round in conformation, and *Passiflora quadrangularis* was irregular conformation.

The cambial variants in Rubiaceae exhibited furrowed or lobed xylem in *Atractogyne* and *Chiococca* respectively (Jansen et al. 2002). In this study we found that *Coptosapelta diffusa, Morinda parvifolia,* and *Morinda umbellata* also possessed this cambial variant type. Jansen et al. (2002) indicated that stem cross section generally develops lignified or unlignified parenchymas in some genera of Rubiaceae. In this study, four genera *Coptosapelta, Mussaenda, Randia,* and *Uncaria* have lignified parenchyma and three genera *Dimetia, Morinda, Paederia* have significant unlignified parenchyma (lianescent habit), these results were consistent with Jansen et al. (2002). The lianescent habit or environmental influences presumably explains the variation in quantitative features or the distribution of axial parenchyma of *M. parvifolia* (Fig. 1D).

The cambial variant of *Cayratia corniculata* (Vitaceae) is a combination of irregular conformation and axial vascular elements in segments in the smaller stem. When the stem size is larger about 3.5 cm in diameter, the second vascular bundles developed near the cortex and will form the successive cambia (Fig. 4F). Pace et al. (2018) indicated *Tetrastigma retinervum* and *Tetrastigma voinierianum* (Vitaceae) had a combination of axial vascular elements in segments with successive cambia, suggesting that the examination of mature stems of additional species of *Tetrastigma* should determine the distribution of this unique cambial variant type. We studied three endemic species, *Tetrastigma formosanum, Tetrastigma hemsleyanum, Tetrastigma lanyuense,* and one native species, *Tetrastigma obtectum* var. *glabru,* showing that only *T. obtectum* var. *glabru* had irregular conformation without successive cambia (Fig. 2C). We speculate that the stem diameter of this species we collected was too small to exhibit successive cambia development.

### Cambial variants of Taiwan lianas in each order

All the differences between the comparisons of the cambial variants of orders/families reported by Angyalossy et al. (2015) are presented in Table 3. Just regular secondary growth of 36 species, belonging to 15 families, 12 orders (Table 3), e.g., *Eleutherococcus trifoliatus* var. *trifoliatus* and *Hedera rhombea* var. *formosana* (Araliaceae) (Table 1) and cambial variants of 16 species, belonging to nine families, and eight orders (Table 3) were added in this study.

In this study, combination types of two or three cambial variants in Cucurbitales were added and the major differences were stems with irregular conformation and furrowed xylem and interxylary phloem. The cambial variants of external secondary vascular cylinder was added and interxylary and intraxylary phloems were not found in Fabales. The cambial variants of Gentianales were diverse and exhibited combinations of two or three cambial variants; only successive cambia was not found. Malpighiales and Myrtales had furrowed xylem of cambium continuity, and furrowed xylem of cambium continuity combined with intraxylary phloem, but four types of cambial variants were not found. Piperales had a combination of external primary vascular cylinders with axial vascular elements in segments, and few species have stem with irregular conformation. In Ranunculales, two combination types were added but irregular conformation, and intraxylary phloem were not found. Six phloem wedges of furrowed xylem of cambium discontinuity are different from four equidistant phloem wedges in Bignoniaceae. Only intraxylary phloem and successive cambia were found in Solanales, but interxylary phloem, furrowed xylems and fissured stem/dispersed xylem were not reported. In Vitales, a combination of three cambial variants was added and only fissured stem/dispersed xylem was not found in Taiwanese species.

The cambial variants types had higher concentration in rosids and asterids (Stevens 2001), and furrowed xylem type was one of the important cambial variants for identification the climbers. The additional information of lianas cambial variants we added here will be available to assess the relationships among the order and families of climbers for academic study.

These reports provide basic data about cambial variants of lianas to establish cambial variants as commonly taxon-specific. The data allows the identification of many individual plants into families or/and genera, even without leaves or flowers.

The developmental processes of stem vascular elements were beneficial to understanding the changes of the vascular bundle of lianas. So in addition to sample larger and freshly stems, one should also sample earlier stages of development in each lianas should be investigated further.

## Conclusion

This study explored the cambial variants of the stem cross-section of Taiwan lianas. The results showed that approximately seven cambial variants and sixteen combination types of cambial variants in 287 Taiwanese lianas were present, highlighting the occurrence of multiple cambial variants of lianas in this region. In most species, axial vascular elements in segments type were exhibited, followed by irregular conformation and intraxylary phloem. Five cambial variants types in Fabaceae and Rubiaceae are the highest, followed in Cucurbitaceae and Vitaceae with four types. Most species in Apocynaceae, Convolvulaceae, and Menispermaceae/ Ranunculaceae had intraxylary phloem, intraxylary phloem combined with successive cambia, and axial vascular element in segments, respectively, showing that these cambial variants could be as a diagnostic characteristics of that family. Comparing the orders/families from the previous data, we added data on the just secondary growth or just vascular bundles of 52 lianas, including 36 lianas with regular secondary growth with rounded stem and 16 lianas with different cambial variants. Approximately 50 remaining Taiwanese lianas are still needed to investigate. Studying the secondary growth, different diameters and corks developments, and the fundamental terms of cambial variants are extremely important for future lianas research. This information on cambial variants of Taiwan lianas will aid in the establishment of cambial variants as commonly taxon-specific and the combination of two or three cambial variants could be a character of taxonomic values, and allow the identification of many individual plants to families or genera without leaves or flowers.
